# Use of rapid detection tests to prevent transfusion-transmitted malaria in India

**DOI:** 10.4103/0973-6247.67033

**Published:** 2010-07

**Authors:** Shalini Bahadur, Meenu Pujani, Manjula Jain

**Affiliations:** Department of Pathology, Lady Hardinge Medical College and Associated Hospitals, New Delhi, India; 1 Blood Bank, Lady Hardinge Medical College and Associated Hospitals, New Delhi, India

Sir,

As we all are aware, transfusion-transmitted malaria is emerging as a major problem especially in developing countries, which fall in the zone of endemicity. Transmission of malaria by blood transfusion was one of the fi rst recorded incidents of transfusion-transmitted infection.[1] The frequency of transfusion-transmitted malaria varies from 0.2 cases per million in nonendemic countries to 50 or more cases per million in endemic areas.[2] Although it was in 1971 that screening of donated blood for viral infections was begun, but there are no defi nite guidelines to the choice of the test. Testing of blood for malarial parasite is mandatory as per the drugs and cosmetic act part X11 B of Schedule F, but there are no clear guidelines to date.

Microscopic detection of blood though considered the gold standard for malaria diagnosis for decades is quite labor-intensive and requires adequate technical skill and manpower. This has spurred the development of several nonmicroscopic malaria rapid detection tests (RDT) based on the detection of malaria parasite antigen in whole blood.

We at our blood bank, Regional Blood Transfusion Centre, have been routinely screening all donated units of blood for malaria using RDT, based on immunochromatographic methods detecting antigens, histidine-rich protein 2 (HRP2-*P. falciparum*), and p-lactate dehydrogenase (pLDH-*P. vivax*) (Paraview, First Sign; Diagnova Pvt. Ltd. New Delhi, India Ltd.) since March 2008. Thick and thin smears were made of all positive cases to corroborate the results of RDT. Total of 11,736 units of donated blood were screened between March 2008 and September, 2009. Three (0.03%) units were found to be positive, 2 for P. vivax, and 1 for *P. falciparum*. All the three cases were found positive microscopically. None of the donor had given a history of fever/malaria during predonation screening [Table 1].

**Table 1:** Comparison of rapid detection tests and peripheral smear screening of positive cases

Case number (positive cases)	Type of malarial parasite	Result of peripheral smear screening	
1	*Plasmodium vivax*	Positive	
2	*Plasmodium falciparum*	Positive	
3	*Plasmodium vivax*	Positive	
**Target antigens for commercially available RDTs**			

	**HRP2**	**pLDH**	**Aldolase**

*P. falciparum* specific	+	+	–
Panspecific (all species)	–	+	+
*P. vivax*	–	*+*	–

The RDT works through lateral flow/immuno chromatographic strip method and signifi es the presence of antigens by a color change on the absorbing nitrocellulose strip. Three main types of antigens are being detected by the commercially available RDTs.

Falade *et al*. in 2009 conducted a study on 391 consecutive potential blood donors in malaria endemic area of South-West Nigeria using 3 methods’ microscopy, OptiMAL RDT (pLDH), and Clinotech Malaria cassette (detects surface protein of merozoites and sporozoites). Microscopy revealed parasitemia in 79 (20.0%) of potential donors, mean level of parasitemia being 445/μl. The corresponding prevalence of malarial parasitemia detected using RDT were 3.8% for OptiMAL and 57.8% for Clinotech, with the results for microscopy being the gold standard.[3]

Bharti *et al*. in 2008 evaluated the usefulness of a new RDT, pLDH/HRP2 malaria card test (First Response Combo Malaria Antigen Test, New Delhi, India) for malaria diagnosis in the forested belt of central India. Analysis revealed that RDT was 93% sensitive, 85% specifi c with a positive predictive value of 79%, and a negative predictive value of 95% (microscopy being used as the gold standard).[4] Kyabayize *et al*. studied the operational accuracy and persistent antigenicity of HRP2 RDTs for Plasmodium falciparum malaria in a hyperendemic region of Uganda. Using a cross-sectional study design, a total of 357 febrile patients of all ages were tested using RDT out of which 40% (139) had positive blood smears for *P. falciparum*. RDT had overall sensitivity of 98%, specifi city of 72%, positive predictive value of 69%, and a negative predictive value of 98%. In the children followed-up after successful antimalarial treatment, the mean duration of persistent antigenicity was 32 days, this duration varied signifi cantly depending on pre-treatment parasitemia.[5] Therefore, a donor who is deferred for 12 weeks following completion of treatment will not give a false positive test for malaria by RDT.

Importance of recognizing transfusion-transmitted malaria lies in the fact that it can lead to febrile transfusion reaction which can falsely simulate a hemolytic transfusion reaction. It can lead to the widespread dissemination and spread of drug-resistant malarial parasite. The utmost importance is derived from the presumption that the screened blood units are transfused to patients ranging from newborns (requiring exchange transfusions) to the geriatric group. These along with the immune-suppressed (cancer and leukemia therapy) patients are at a higher risk of falling prey to units not effectively screened for malarial parasite. Thus clinicians will waste valuable time manage on lines of hemolytic transfusion reaction. Nonetheless, no matter what strategy is adopted, it is likely that cases of transfusion-transmitted malaria may still occur, so malaria must always be considered in any patient with a febrile illness post-transfusion.

Thus we would like to recommend that use of rapid detection devices with peripheral smear screening of positive cases is reasonably reliable method to prevent transfusion-transmitted malaria in India.
